# In Vitro Gingival Wound Healing Activity of Extracts from *Reynoutria japonica* Houtt Rhizomes

**DOI:** 10.3390/pharmaceutics13111764

**Published:** 2021-10-22

**Authors:** Izabela Nawrot-Hadzik, Adam Matkowski, Artur Pitułaj, Barbara Sterczała, Cyprian Olchowy, Anna Szewczyk, Anna Choromańska

**Affiliations:** 1 Department of Pharmaceutical Biology and Biotechnology, Division of Pharmaceutical Biology and Botany, Wroclaw Medical University, 50-556 Wroclaw, Poland; bbsekret@umed.wroc.pl; 2 Department of Dental Surgery, Wroclaw Medical University, 50-425 Wroclaw, Poland; artur.pitulaj@umed.wroc.pl (A.P.); barbara.sterczala@umed.wroc.pl (B.S.); cyprian.olchowy@umed.wroc.pl (C.O.); 3 Department of Molecular and Cellular Biology, Faculty of Pharmacy, Wroclaw Medical University, 50-556 Wroclaw, Poland; a.szewczyk@umw.edu.pl (A.S.); anna.choromanska@umed.wroc.pl (A.C.); 4 Department of Animal Developmental Biology, Institute of Experimental Biology, University of Wroclaw, 50-328 Wroclaw, Poland

**Keywords:** *Reynoutria*, *Polygoni cuspidati rhizoma*, Japanese knotweed, gingival wound healing, resveratrol, proanthocyanidins

## Abstract

Rhizomes of *Reynoutria japonica* Houtt. are a traditional Chinese medicinal herb (*Polygoni cuspidati rhizoma*, hu zhang) used for treatment of numerous diseases including wound healing support. The aim of this study was to provide evidence for the value of this herbal drug’s traditional use as a gingival healing treatment as well as to obtain the most active extract. In vitro studies were performed using primary human gingival fibroblasts (HGFs) with determination of viability (MTT assay), cell proliferation (the confocal laser scanning microscope (CLSM) was used to visualize histone 3 expression), cell migration (wound healing assay), and evaluation of the expression of collagen type III (immunocytochemical staining) after incubation with extracts from *R. japonica* rhizomes (25% or 40% ethanol or 60% acetone). In addition to these extracts, commercial dental rinse (containing chlorhexidine digluconate 0.2%) was tested as the gold standard of choice for gum healing in dental practice. The studied extracts were qualitatively and quantitatively characterized using the validated HPLC/DAD/ESI-HR-QTOF-MS method. Total phenols and tannins content were determined using the Folin–Ciocalteu assay. Low concentration of all extracts after 24 h incubation caused significant increase in HGF viability. This effect was most pronounced at a concentration of 50 µg/mL, which was selected for further experiments. All extracts (at 50 µg/mL) stimulated HGF to proliferate, migrate, and increase collagen III synthesis, but with different strength. The highest stimulated proliferation and migration activity was observed after incubation with 25% EtOH, which according to phytochemical analysis may be related to the highest content of resveratrol and an appropriate composition of procyanidins. The 25% EtOH extract from *R. japonica* rhizomes appears to be a promising gingival wound healing agent worthy of animal and clinical trials.

## 1. Introduction

Rhizomes of *Reynoutria japonica* Houtt. (syn. *Fallopia japonica* (Houtt.) Ronse Decr., *Polygonum cuspidatum* Sieb. & Zucc.; placed within the family *Polygonaceae,* genus *Fallopia*, section *Reynoutria,*) are a traditional Chinese medicinal herb (known in pharmacopoeias as *Polygoni cuspidati rhizoma* and as hu zhang in *pinyin* Chinese) that have long been used for treatment of numerous diseases, such as hyperlipemia, inflammation, infection, and cancer, etc. [1]. This plant is listed in many monographs of traditional Chinese medicine, starting from the Mingyi Bielu, written in China during the Han dynasty, about 1800 years ago [1]. In later monographs of Materia Medica: Dian-nan Bencao, Rihuazi Bencao, Sichuan Zhoanyaozhi and others, rhizomes of this plant were mentioned to be used for treatment of suppuration, sore throat, toothache, ulcer, and other ailments [1]. In the modern traditional Chinese medicine monograph [2], the broad spectrum of activity of this plant was mentioned, inter alia: indication in injuries, skin infections, inflamed wounds (internal and external). Moreover, in Korean folk medicine, this plant is used to improve oral hygiene [3]. The traditional use of *R. japonica* in wound healing has only been tested in one animal study so far. A 50% methanolic extract of *R. japonica* rhizomes applied topically to the skin accelerated the wound healing process in rats [4]. In the present study, we sought to provide evidence to explain the traditional use of *R. japonica* in wound healing, primarily in the context of its use in the oral cavity. We based the study on our earlier research [5], in which we showed that 70% acetone extract from rhizomes of *R. japonica* demonstrated antibacterial activity against dominant caries pathogen-*Streptococcus mutans* and additionally increased viability of human gingival fibroblasts (MTT assay, up to 138% compared with the control) after 24 h incubation with low concentrations of extracts (from 5 to 250 μg/mL). However, as the MTT reduction is a marker reflecting viable cell metabolism, not specifically cell proliferation [6], in the current experiment we studied proliferative activity of gingival fibroblasts using confocal laser scanning microscopy (CLSM). Gingival fibroblasts, the most abundant cell type in connective tissue, play a significant role in healing wounds inside the oral cavity. Through proliferation and differentiation, they contribute to the formation of new tissues and their remodeling. They synthesize and secrete type III collagen, which is resorbed over time and replaced by type I collagen. Because of a high expression of type III collagen during wound healing [7,8], we also checked the effect of the extracts on collagen III synthesis.

Oral wound healing agents are desirable in patients whose natural healing process is impaired, such as diabetic patients suffering from periodontal disease in which high glucose level and high glucose-induced oxidative stress level impairs the proliferation and migration of HGFs [9]. Such healing agents are also desirable for use in patients after chemotherapy and radiotherapy who are suffering from oral mucositis [10], as well as in patients suffering from immunological disorders and systemic diseases [11]. Moreover, old age also predisposes to worse wound healing due to, e.g., impairment in the proliferation, migration, and differentiation of fibroblasts and reduction in the synthesis of type I and III collagen [12]. Since wounds in the oral cavity are prone to infectious processes, mainly of a multi-microbial nature, which impede the healing process, it is recommended to use antiseptics or agents that promote the tissue repair process [13]. Chlorhexidine is one of the most commonly used antiseptic wound healing agents. Despite its proven antimicrobial activity [14], it has side effects, such as temporary loss or impairment of taste as well as discoloration all over the dentition [15]. Moreover, in vitro studies revealed its cytotoxic effect on fibroblasts, which may adversely affect the healing process [13]. That is why we also included commercial dental rinse (containing chlorhexidine digluconate 0.2%) in our research. In addition to the acetone extract of *R. japonica* rhizomes, which in earlier studies showed an increase in cell viability, we also tested ethanol extracts to obtain the best combination of compounds that can be used in the future in dental practice. The qualitative (HPLC/DAD/ESI-HR-TOF-MS) and quantitative analysis of extracts revealed the differences between them, which allowed our nomination of compounds responsible for the observed activity.

## 2. Materials and Methods

### 2.1. Plant Material and Extract Preparation

Rhizomes of *Reynoutria japonica* used in this study were collected during the first week of October 2020. Rhizomes were harvested in urban habitats near the city of Wroclaw (Poland): 51°07.404′ N 17°04.146′ E. Only fully developed and undamaged rhizomes with a diameter of 15–30 mm were harvested. Klemens Jakubowski (MSc Botany) from Botanical Garden of Medicinal Plants herbarium identified the species. The voucher specimens H-001 were deposited in the Botanical Garden of Medicinal Plants, Medical University of Wroclaw. The plant name-*Reynoutria japonica* Houtt. (syn. *Fallopia japonica* (Houtt.) Ronse Decr., *Polygonum cuspidatum* Sieb. & Zucc.) was checked with http://www.plantsoftheworldonline.org (access to this website: 6 July 2021).

Fifty grams of air-dried and powdered rhizomes of *R. japonica* were extracted with 500 mL of 25% ethanol or 40% ethanol or 60% acetone (each extraction with ultrasonic bath, 2 h). The solvent was evaporated under reduced pressure. Stock solutions were prepared from the dried extracts by dissolving 100 mg of extract in 1 mL of DMSO. By taking an appropriate amount of the stock solution, different concentrations of extracts (5–2000 µg/mL) were investigated. As a solvent to prepare different concentrations of extracts, the cell culture medium was used.

### 2.2. HPLC/DAD/ESI-HR-QTOF-MS Qualitative and Quantitative Analysis

All the obtained extracts were prepared for the qualitative and quantitative analysis. An amount of 50 mg of dried extracts was dissolved in 80% MeOH in volumetric flasks to obtain 5 mg/mL concentration. Then the solutions were filtered through a 0.22 µm Chromafil syringe membrane (Macherey-Nagel, Düren, Germany) to autosampler vials and were injected (4 µL) to the High-Pressure Liquid Chromatography (HPLC) system.

#### 2.2.1. HPLC Apparatus

The Ultimate 3000RS series system (Thermo Dionex, Sunnyvale, CA, USA) equipped with a low-pressure quaternary gradient pump was used, with vacuum degasser, an autosampler, a column compartment, a DAD, and a high-resolution quadrupole time-of-flight MS (Bruker qTOF Compact, Bruker Daltonik, Billerica, MA, USA) equipped with ESI. The system was controlled by Bruker Compass Hystar software (Billerica, MA, USA).

#### 2.2.2. HPLC-DAD-MS Conditions

Column: analytical Kinetex C18 2.6 µm (150 mm × 2.1 mm), (Phenomenex, Torrance, CA, USA) maintained at 30 °C. Mobile phase A (H_2_O:HCOOH, 100:0.1, *v/v*) and B (acetonitrile:HCOOH, 100:0.1, *v/v*) were used in a following gradient program: 0–22 min 15–22% B, 22–33 min 22–95% B, followed by column equilibration with 15% B for 2 min between injections. The flow rate was 0.3 mL/min. Analysis of all samples was repeated four times as consecutive injections. UV-Vis spectra were recorded in the range of 200–450 nm. Chromatograms were acquired at 298 nm. High resolution quadrupole time-of-flight mass spectrometer was equipped with electrospray ionization (ESI-HR-QTOF-MS). ESI-MS conditions were as follows: splitless, nebulizer pressure 30 psi; dry gas flow 8 L/min; dry temperature 250 °C; and capillary voltage 2.2 kV for negative ion mode and 4.5 kV for positive ion mode (for physcion). Mass spectra were recorded using scan range (*m*/*z*) 50–2200. The collision energy was set automatically from 20 to 40 eV, depending on the *m*/*z* of the fragmented ion.

The quantitative analysis of obtained extracts was performed as described in our previous paper [16].

### 2.3. Total Polyphenols and Tannins Content

A modified Folin–Ciocalteu assay based on Singleton and Rossi method [17], was used for determination of total polyphenol content as described in our previous studies [18,19]. Tannin compounds were measured by parallel experiments with extracts vortexed for 1 h with 10 mg/mL hide powder. The results were expressed as gallic acid equivalents according to the standard gallic acid calibration curve. Total tannins were calculated by subtraction of polyphenols non-absorbed by hide powder from the total polyphenol content.

### 2.4. Cell Culture

The experiments were conducted on a primary human gingival fibroblast cell line. The experimental protocol was accepted by the Bioethics Commission of Wrocław Medical University, No. KB-434/2017. The tissue cultures of human gingival fibroblasts were extracted from healthy adult volunteers. The gingival biopsy was provided by the Department of Dental Surgery at Wrocław Medical University. The epithelial-connective tissue fragment was remoted from the hard palate using the “punch” method. This method allows the connective tissue layer located closest to the epithelium to be obtained, which is characterized by effective keratosis. Cells were maintained at 37 °C, 5% CO_2_, 95% air humidity in DMEM medium (Sigma-Aldrich, Poznan, Poland) supplemented with 10% fetal bovine serum (Sigma-Aldrich, Poznan, Poland). Cell passages were carried out once a week when confluency was about 90%. Cells were removed from the culture vessel by trypsinization (trypsin 0.25% and EDTA 0.02%; Sigma-Aldrich, Poznan, Poland).

### 2.5. Cell Viability Assay

The MTT assay (Sigma-Aldrich, Poland) was used to assess fibroblast cell viability. The cellular metabolic activity test (MTT) is based on the reduction of tetrazolium salts by metabolically active cells. By the action of dehydrogenase enzymes, reducing equivalents such as NADH and NADPH are generated. Intracellular purple formazan is solubilized and quantified by spectrophotometry. The colored solution was quantified by measuring absorbance at 570 nm using a multi-well spectrophotometer (GloMax^®^ Discover multimode microplate reader, Promega, Walldorf, Germany). The mitochondrial metabolic function was represented as a percentage of viable treated cells to untreated cells. The extracts were added at concentrations of 5–2000 µg/mL dissolved in dimethyl sulfoxide (DMSO, Sigma-Aldrich, Poland). DMSO concentration in the samples with the highest solution was 2%. The control sample with 2% DMSO was also included in the MTT test. For comparison with an agent routinely applied in oral hygiene and treatment, we used the commercial mouth rinse “Corsodyl 0.2%” (GlaxoSmithKline, containing chlorhexidine digluconate 0.2% *w*/*v* as active agent and other ingredients: glycerol, macrogolglycerol hydroxystearate, sorbitol liquid (non-crystallizing), Optamint aroma 291,616, and purified water). The MTT test was performed on 96-well plates, where 1 × 10^4^ cells were seeded for one well. The estimation of viability was carried out after 5 min, 6 h, and 24 h. All samples were analyzed in six replicates.

### 2.6. Confocal Laser Microscopy Study

A confocal laser scanning microscope (CLSM, Olympus FluoView FV1000, Tokyo, Japan) was used to visualize histone 3 expression change after incubation with 50 µg/mL extracts and “Corsodyl 0.2%” (containing chlorhexidine digluconate 0.2% *w*/*v* as active agent). Cells were harvested on cover glasses in Petri dishes overnight, then the medium was replaced by test medium (containing the extracts or pure DMSO) and incubated at 37 °C for 24 h. Afterwards, the samples were washed in PBS and fixed in 4% formaldehyde. Then, the cells were permeabilized with 1% Triton X-100 in PBS for 5 min, raised in PBS 3 × 5 min, blocked with 4% bovine serum albumin (BSA) in PBS for 1 h, and the primary rabbit polyclonal antibody anti-phospho-histone H3 (Ser10; Millipore, Burlington, MA, USA), 06-570) at a concentration of 1:200 was added and left for 2 h. Samples were washed with PBS three times and incubated with the secondary goat anti-mouse antibody, Alexa Fluor 488 (A-11029, Invitrogen, Waltham, MA, USA) at a concentration of 1:500 for 1 h at 37 °C. After the incubation, the cells were washed in PBS, and the samples were mounted in fluorescence mounting medium with DAPI (DAKO) to visualize nuclei. Images were taken using CLSM with an oil immersion objective lenses in 60× magnification. The intensity of fluorescence of histone H3 after 24 h incubation with different extracts of *Reynoutria japonica* in fibroblasts was determined by ImageJ analysis software (NIH, Bethesda, MD, USA).

### 2.7. In Vitro Wound Healing Assay

Effect of extracts at 50 µg/mL and the 1.56% solution of dental rinse (“Corsodyl 0.2%”, containing chlorhexidine digluconate 0.2% *w*/*v* as active agent) on fibroblasts’ motility was investigated using a cell migration-based wound healing assay. The most characteristic readout of this in vitro wound healing assay is the change of the cell-covered area (gap closure) over the period of time. The Culture-Insert2-Well (Ibidi, Gräfelfing, Germany) was provided with two reservoirs for culturing cells that were separated by a 500 μm thick wall. The cells were seeded in the reservoirs and cultured until they attached and formed a monolayer. Removal of the silicone insert from the surface resulted in two precisely defined cell patches, which were separated by a zone that had exactly the same width as the separation wall. Cell migration was monitored by taking photos at different time points. Images were captured directly after insert removal and after 24, 48, and 72 h of observation using a Leica DMi1 light microscope (Wetzlar, Germany).

### 2.8. Immunocytochemical Staining

Immunocytochemical staining was used for semiquantitative evaluation of the expression of collagen type III after 24 h incubation with extracts at 50 µg/mL or 1.56% solution of the dental rinse (“Corsodyl 0.2%”, containing chlorhexidine digluconate 0.2% *w*/*v* as active agent). For positive control, betulinic acid (2 µM) was also used. Cells were trypsinized and suspended in a culture medium with the addition of 50 µg/mL tested extracts, commercial dental rinse, or betulinic acid. Cultures were maintained on 10-well microscopic diagnostic slides (Thermo Scientific, Waltham, MA, USA) and incubated for 24 h. Then slides were fixed using 4% paraformaldehyde for 10 min. To execute immunocytochemical staining, the Mouse and Rabbit Specific HRP/DAB Detection IHC kit (Abcam, Cambridge, UK) was used. After rinsing in PBS, any remnant peroxidase activity was removed by incubation with hydrogen peroxide block for 10 min. Stained cells were permeabilized by incubation with 1% Triton X 100 (Sigma-Aldrich, Poland) and exposed overnight to a primary antibody: rabbit polyclonal IgG (dilution rate: 1:200, Anti-Collagen III antibody, ab7778, Abcam, Poland) at 4 °C. Then, the secondary antibody conjugated with horseradish peroxidase (HRP) was added to samples. For visualization, the reaction samples were incubated with diaminobenzidine–H_2_O_2_ mixture. Hematoxylin (Alchem, Wroclaw, Poland) was used for cell nuclei staining. Slides were dehydrated using ethanol gradient and covered using DPX gel (Aqua-Med Zpam-Kolasa, Łódź, Poland). An upright microscope (Olympus BCX43) was used to examine the immunocytochemical reaction. Immunocytochemistry analysis was performed using the ABC method [20]. The intensity of immunohistochemical staining was evaluated as (−) negative, (+) weak, (++) moderate, or (+++) strong.

### 2.9. Statistical Analysis

All the experimental procedures were performed at least in triplicate. The values obtained from the MTT assay are presented as the mean of six replicates ± SD. To evaluate the distribution of the results, the Shapiro–Wilk test was used. Significant differences between mean values were evaluated by two-way ANOVA and Tukey’s multiple comparisons test using GraphPad Prism v. 9 (GraphPad Software, San Diego, CA, USA). The *t*-test was used for the means comparison between treatments and the control.

## 3. Results and Discussion

### 3.1. Cell Viability—MTT Assay

**Figure 1:**
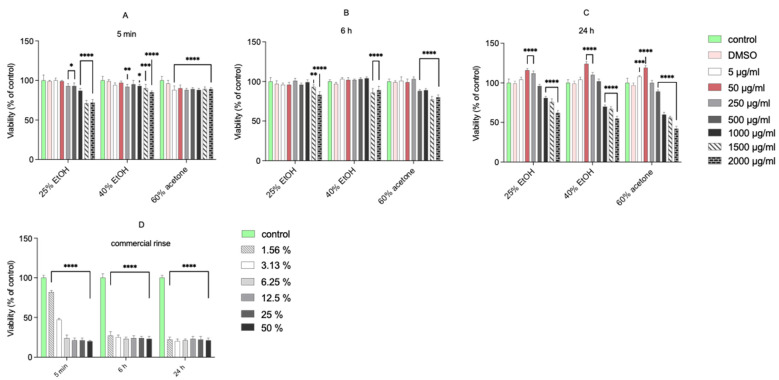
Viability of fibroblast cell line after 5 min (**A**), 6 h (**B**), and 24 h (**C**) incubation following increasing concentrations of different extracts of *Reynoutria japonica* and commercial rinse (**D**). Note that 25% EtOH, 40% EtOH, and 60% acetone means 25% ethanol extract, 40% ethanol extract, or 60% acetone extract from *Reynoutria japonica* rhizomes, respectively. The mitochondrial metabolic function (viability) is expressed as a percentage of viable treated cells in relation to untreated control cells. Error bars shown in this figure are means ± SD for *n* ≥ 6. On the graphs, the statistical differences between the mean of the treatments and the control sample are marked. * Statistically significant at *p* ≤ 0.05, ** for *p* ≤ 0.01, *** for *p* ≤ 0.001, **** for *p* ≤ 0.0001.

**Figure 2:**
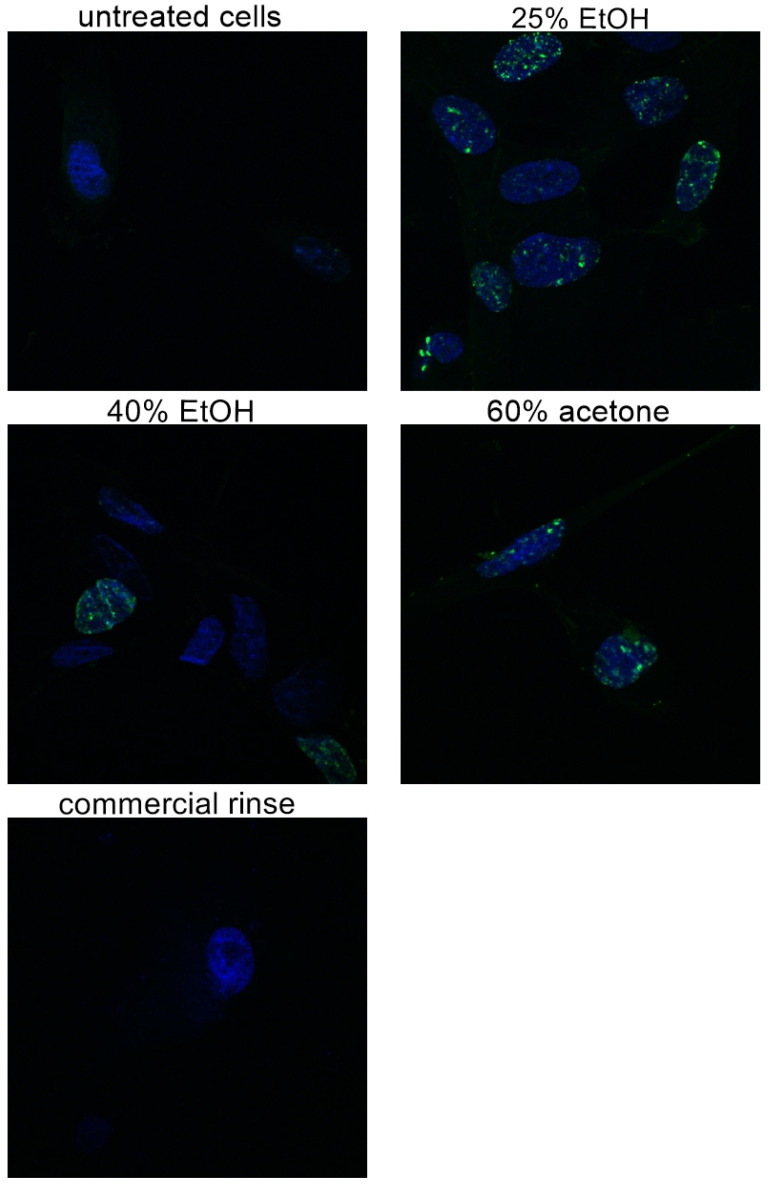
Microphotographs of fibroblasts with immunofluorescence staining histone H3 after 24 h incubation with different extracts of *Reynoutria japonica* (at the concentration of 50 µg/mL) and commercial dental rinse (1.56% solution). Note that 25% EtOH, 40% EtOH, and 60% acetone means 25% ethanol extract, 40% ethanol extract, or 60% acetone extract from *Reynoutria japonica* rhizomes, respectively. Magnification on panel: ×600. Alexa Fluor 488 used for histone-3 labeled, DAPI used for the cell nucleus labeled.

The tested extracts were not cytotoxic to normal human gingival fibroblasts at concentrations from 5 to 500 μg/mL, following a 5 min, 6 h, or 24 h incubation (Figure 1). Only the highest concentrations (from 1000 μg/mL to 2000 μg/mL) of extracts significantly decreased the viability of fibroblasts. The commercial mouth rinse was markedly toxic to the cells even at the lowest applied concentration (1.56%). In contrast, after 24 h incubation of fibroblasts with low concentrations of extracts, a significant increase in cell viability was seen (up to 124% compared with the control for 50 μg/mL of 40% EtOH extract). These results are in accordance with our previous report [5], where incubation of normal human gingival fibroblasts with 50 or 250 μg/mL of 70% acetone extract caused a significant increase in their viability. This observation suggests a stimulating effect on the regenerative potential of fibroblasts. However, the MTT reduction reflects viable cell metabolism, which is only an indirect marker of cell proliferation [6]. To verify whether the living cells proliferate upon treatment, we used the confocal laser scanning microscope to visualize histone 3 expression change after incubation with extracts (at 50 µg/mL) and commercial dental rinse (Figure 2).

### 3.2. Confocal Laser Microscopy Study

**Figure 3:**
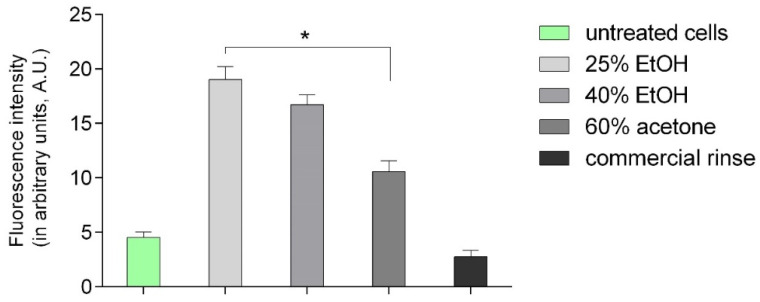
Intensity of fluorescence of histone H3 after 24 h incubation with different extracts of *Reynoutria japonica* in fibroblasts in arbitrary units. Error bars shown in this figure are means ± SD for *n* ≥ 5. * statistically significant at *p* < 0.0001.

Figure 2 presents microphotographs of fibroblasts with immunofluorescent staining of histone H3 after 24 h incubation with different extracts of *Reynoutria japonica* (at 50 µg/mL) and commercial dental rinse (1.56% solution). The primary rabbit polyclonal antibody anti-phospho-histone H3 was used to present abundance of phosphorylation of serine 10 on histone (H3S10ph). As the phosphorylation of serine 10 on histone 3 increases during cell division [21,22], the influence of studied extracts on proliferation of fibroblasts could be determined. As shown above (Figure 2 and Figure 3), in contrast with commercial rinse and untreated cells, all tested extracts stimulated human gingival fibroblasts to divide. The most intense H3S10ph was observed after incubation with 25% EtOH extract.

### 3.3. Wound Healing Assay

**Figure 4:**
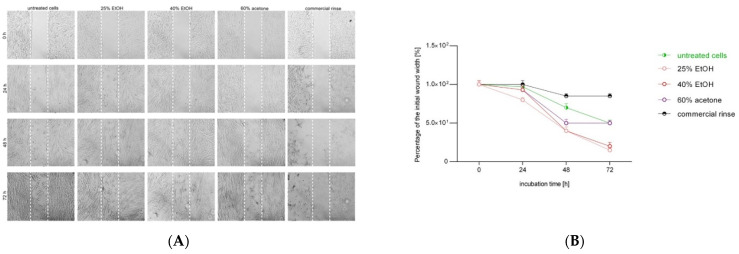
Effect of different extracts of *Reynoutria japonica* (at 50 µg/mL) and commercial dental rinse (1.56% solution) on fibroblast motility investigated with wound healing assay. Note that 25% EtOH, 40% EtOH, and 60% acetone means 25% ethanol extract, 40% ethanol extract, or 60% acetone extract from *Reynoutria japonica* rhizomes, respectively. (**A**) Images of wound gradually invaded by migrating cells; (**B**) The percentage of a healed wound as a function of time. Images were analyzed by ImageJ software; images were taken in given time intervals. The graph represents the data form the three replicates of an individual experiment. Data are mean ± SD (*n* = 3 replicates).

Bearing in mind the possibility of using preparations stimulating the division of fibroblasts in the treatment of wounds, we carried out the wound healing assay with the use of all extracts (at 50 µg/mL) and the commercial dental rinse (1.56% solution). Results are presented in Table 1 and Figure 4.

Wound healing assays are a widely used approach for the analysis of cell migration under different conditions. The most characteristic readout of a wound healing assay is the change of the cell-covered area (gap closure) over time. As presented in Figure 4, all tested extracts showed wound healing activities higher than those of the control group and commercial rinse. The results suggest that 25% EtOH extract and 40% EtOH extract display slightly higher cell migration than that of 60% acetone extract. Moreover, the advantage in migration of 25% EtOH extract was already visible after 24 h of incubation.

### 3.4. Immunocytochemical Staining

**Figure 5:**
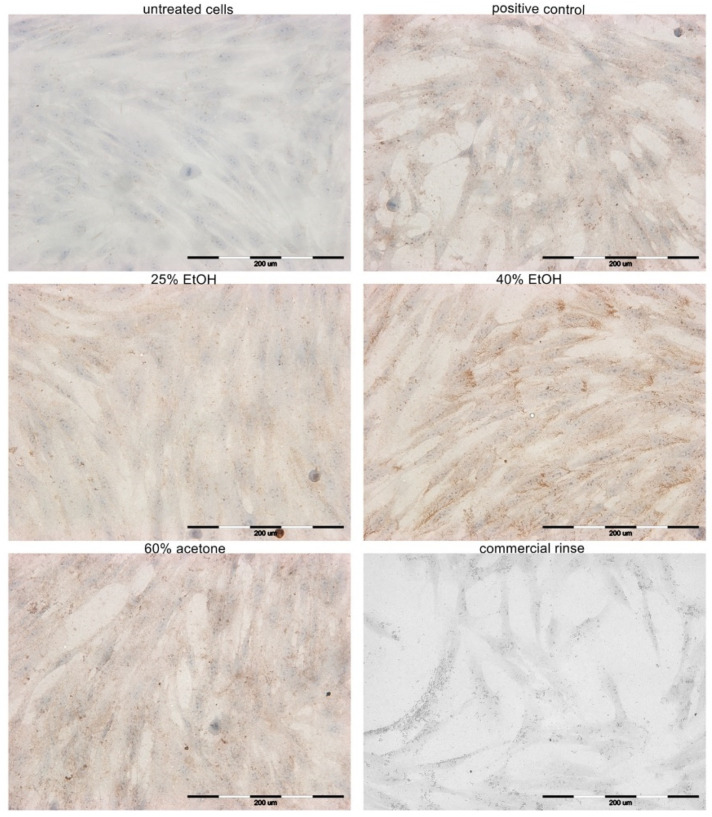
Microphotographs of fibroblasts with immunocytochemically stained collagen type III after 24 h incubation with different extracts of *Reynoutria japonica* (at 50 µg/mL) and commercial dental rinse (1.56% solution). Note that 25% EtOH, 40% EtOH, and 60% acetone means 25% ethanol extract, 40% ethanol extract, or 60% acetone extract from *Reynoutria japonica* rhizomes, respectively. Positive control: betulinic acid (2 µM). Magnification on panel: ×200.

During the wound healing process, the production of collagen type III by fibroblasts plays a significant role. Figure 5 presents the results of experimental collagen type III evaluation in primary HGFs. Table 2 presents semi-quantitative results.

The results of the expression of collagen type III in primary HGFs were determined after 24 h of incubation with different extracts of *Reynoutria japonica* (at 50 µg/mL), commercial dental rinse (1.56% solution), and betulinic acid (2 µM) as positive control. A similar increase in collagen type III quantity was observed in HGFs after incubation with positive control and 60% acetone extract. Higher intensity of immunocytochemical reaction was observed after incubation with the 40% EtOH extract, but lower intensity was observed after incubation with the 25% EtOH extract. Incubation HGFs with commercial rinse revealed a lack of expression of this protein, similar to the untreated cells.

### 3.5. HPLC/DAD/ESI-HR-TOF-MS Qualitative and Quantitative Analysis

**Figure 6:**
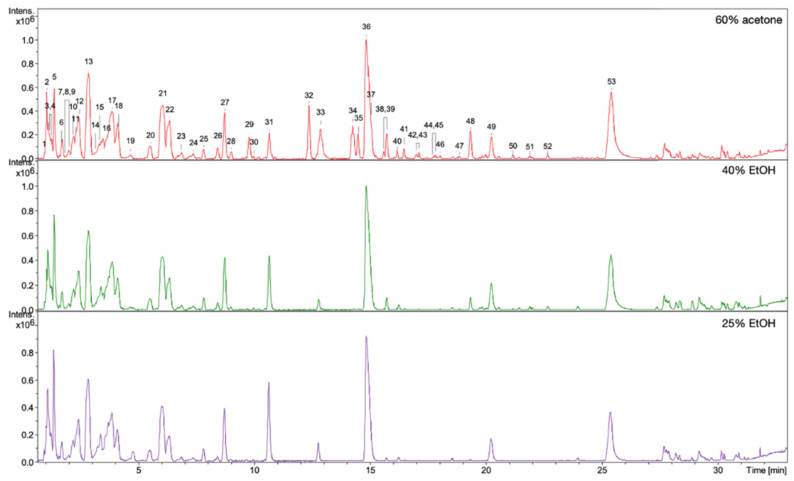
ESI-MS (negative mode) chromatograms of 60% acetone, 40% EtOH, and 25% EtOH extracts from rhizomes of *R. japonica*. Key to peak identity as in Table 3.

**Figure 7:**
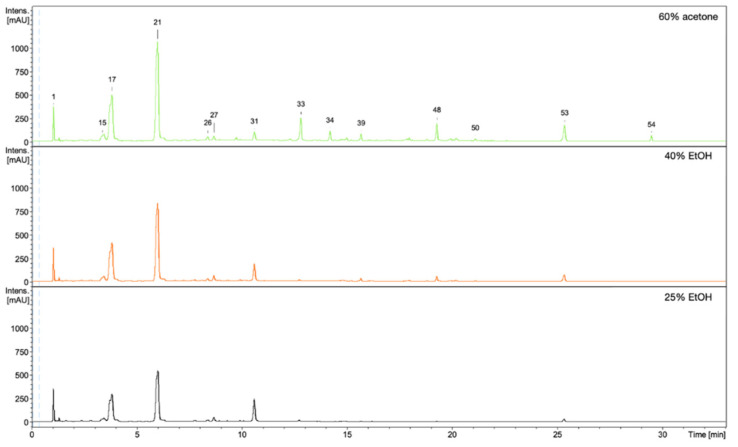
UV-HPLC chromatograms of 60% acetone, 40% EtOH, and 25% EtOH extracts from rhizomes of *R. japonica* with detection at 298 nm.

The observed stimulating effects of the extracts on the proliferation, migration of fibroblasts, and on the production of collagen III depend on the composition of the compounds in the extracts. Qualitative and quantitative HPLC/DAD/ESI-HR-QTOF-MS analyses were performed for all extracts in order to determine the composition of the compounds and the content of some of them. The UHPLC-QTOF-MS analysis of 60% acetone, 40% EtOH, and 25% EtOH extracts from rhizomes of *R. japonica* revealed a total of 54 different compounds (Table 3) belonging to stilbenes, carbohydrates, procyanidins, flavan-3-ols, anthraquinones, phenylpropanoid disaccharide esters, hydroxycinnamic acids, naphthalenes, and their derivatives. Most of the compounds were earlier tentatively identified in extracts and fractions from rhizomes of *R. japonica* and were described in our previous papers [23,24], but in this study, a high-resolution mass spectrometer was used, which allowed full confirmation of the earlier observations. Although the ESI-MS (Figure 6) and UV-HPLC (Figure 7) chromatograms of all extracts seem very similar in quality, there are differences, especially in quantity of compounds. Some of the compounds—mainly those belonging to anthraquinones and phenylpropanoids (corresponding peak numbers in the parentheses): emodin-glucoside (29), torachrysone-hexoside (32), emodin-glucoside (33), emodin-8-O-(6′-O-malonyl)-glucoside (34), unknown torachrysone derivative (35), emodin bianthrone-hexose-(malonic acid)-hexose (38), hydropiperoside (39), and vanicoside B (48)—were abundant only in the 60% acetone extract, while being observed only in trace amounts in the 40% EtOH or in 25% EtOH.

The differences in the amount of some compounds in the extracts were revealed by the use of the previously developed and validated analytical method [16] for determination of six main compounds from three phytochemical classes—stilbenes: piceid and resveratrol; anthraquinones: emodin and physcion; phenylpropanoid disaccharide esters: vanicosides A and B. The 60% acetone extract contained the highest amount of almost all quantified compounds, except for resveratrol (Table 4 and Table 5). A significantly lower amount of the determined compounds was in the 40% EtOH extract, and the lowest amount was in the 25% EtOH extract. Interestingly, resveratrol showed the opposite trend. The highest amount was found in the 25% EtOH and the lowest in the 60% acetone extract.

The above difference in the composition of the tested extracts is consistent with the principle of dissolution of chemical compounds in solvents of similar polarity. Hence, in acetone extract, in a solvent less polar than ethanol, we observed more substances of lower polarity [25]. A similar relationship can be observed in the more polar 25% ethanol extract and the less polar 40% ethanol extract. However, the reverse tendency of resveratrol is surprising, the solubility of which, according to the literature in ethanol + water mixtures, should increase with the increase of the ethanol molar fraction. The explanation of this phenomenon requires more detailed research. However, other compounds present in the 25% ethanol extract may affect the better dissolution of resveratrol (by means of the so-called co-solubility or a putative matrix effect), as well as other factors, such as the significant effect of water on tissue swelling, which can increase ethanol penetration into the plant tissue [26,27].

Considering that 60% acetone extracts did not show a better stimulation of human gingival fibroblasts to division, migration, and collagen III synthesis than ethanol extracts, it seems that the higher content of abovementioned compounds observed in that extract than in ethanol extracts did not affect this activity. Instead, paying attention to resveratrol should be made. Its content is higher in ethanol extracts, especially in 25% ethanol extract, which correlates with stronger stimulation of fibroblasts to proliferate and migrate. Resveratrol is a well-researched compound with established biological activity, including antioxidant, anti-angiogenic, anti-inflammatory, and antimicrobial properties, as well as cytotoxic activity against cancer cell lines and with a positive effect on the cardiovascular system and others [28]. Rhizomes of *Reynoutria japonica* are the one of the richest natural sources of resveratrol [1]. Neither of the closely related species (*Reynoutria sachalinensis* [F.Schmidt] Nakai, *Reynoutria* × *bohemica* Chrtek & Chrtková, family *Polygonaceae*) possess so high an amount of this compound [16]. The results from our earlier studies showed that among three plant extracts from different *Reynoutria* species—*R. japonica*, *R. sachalinensis*, *R.* × *bohemica—*only *R. japonica* extract caused increased viability of normal human fibroblasts after 24 h incubation. Moreover, in the study of Chin et al. [29], enhanced cell proliferation of human gingival fibroblasts was observed after incubation with low concentration (10 µM) of resveratrol but inhibited cell proliferation was observed at higher concentrations (100 and 200 µM). Thus, resveratrol could have an impact on the stronger stimulation activity of 25% EtOH extract to proliferate fibroblasts than the other two extracts. In addition to the mentioned stilbenes, anthraquinones, and phenylpropanoid disaccharide esters, the HPLC/DAD/ESI-HR-QTOF-MS analysis revealed that all extracts contain significant amount of procyanidins, which according to the literature may affect the proliferation of fibroblasts [30,31]. To show the differences in the content of these compounds in the extracts, the total polyphenols and tannins content was examined.

### 3.6. Total Polyphenols and Tannins Content

**Figure 8:**
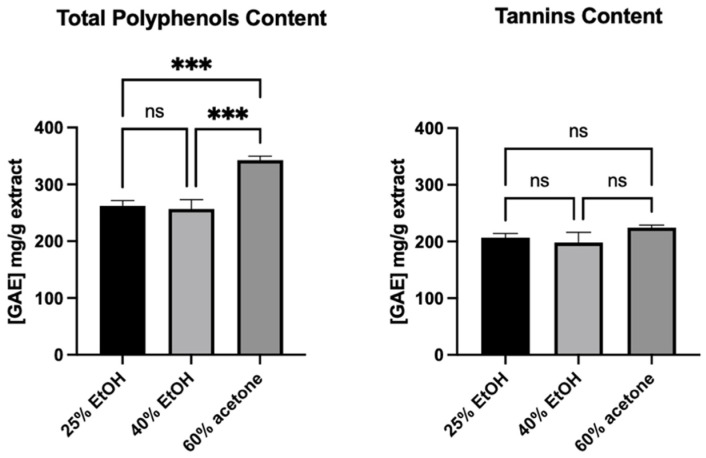
Total polyphenols and tannins content in studied extracts. Note that 25% EtOH, 40% EtOH, and 60% acetone means 25% ethanol extract, 40% ethanol extract, or 60% acetone extract from *Reynoutria japonica* rhizomes, respectively Error bars shown in this figure are means ± SD for *n* ≥ 3. *** Statistically significant at *p* ≤ 0.001, ns: not statistically significant.

Although the 60% acetone extract showed a higher total polyphenols content than the ethanol extracts, which is in accordance with HPLC/DAD/ESI-HR-QTOF-MS analysis, the tannins content was the same for all of them (Figure 8). Moreover, tannins made up most of the polyphenols in the extracts.

As the 60% acetone extract did not show any stronger stimulating effect than the other two, it seems that the observed greater amounts of other polyphenols than tannins did not affect this activity. The study of Wang R. et al. [30] showed that tannin-rich extract and fractions from the root bark of *Paeonia suffruticosa* Andrews (family *Paeoniaceae*) (a TCM herb) enhanced cell viability and cellular proliferation of human primary dermal fibroblasts (pNHDF) and HaCaT keratinocyte (after 48 h incubation time). The isolated tannins were tested, and it was proved that hydrolysable and condensed tannins are responsible for cell stimulation. The stimulating effect on fibroblasts and keratinocytes was observed at low concentrations, while higher concentrations inhibited cell proliferation. Based on this evidence, it can be concluded that the tannins observed in our *R. japonica* extracts may have an effect on the observed increased viability and proliferation of human gingival fibroblasts. Importantly, literature data on the extraction of procyanidins from raw materials using various concentrations of ethanol or acetone mixtures reveal significant differences in the composition of tannins [32]. In a study by Downey et al. [32], the average length of the tannin polymers extracted from grape skin with ethanol mixtures was significantly lower than in the acetone mixtures (11.3 subunits in the 20% EtOH, 15.8 subunits in 40% EtOH, and 27.2 subunits in 60% acetone). It is likely that this variation occurs in our extracts, and it might not have influence on observed total tannins content measured by the Folin–Ciocalteu methods [19], which are based on the reducing properties of compounds. Thus, the differences in the composition of procyanidins could affect the observed differences in the activities of extracts. Moreover, according Kisseih et al. [33], even a slight difference in the structure of procyanidins can change their stimulating effect on cell proliferation. In their study, procyanidin B2 (epicatechin-(4β-8)-epicatechin) stimulated cellular differentiation of primary keratinocytes, but procyanidin B5 (epicatechin-(4β-6)-epicatechin), different only in the interflavan linkage between both epicatechin blocks, did not. This indicates a specific interaction of procyanidin dimer with a potential molecular target rather than nonspecific astringent “tannin-like” effects.

The obtained 25% EtOH extract seems to be a good choice for further animal studies and clinical trials. It is stronger than other extracts and stimulated fibroblasts proliferation and migration, which may be related to its highest content of resveratrol and the appropriate composition of procyanidins in it. Both resveratrol and procyanidins have proven immunomodulatory, anti-inflammatory, antioxidant, and antibacterial activity, positively influencing the prevention or treatment of periodontal diseases [34,35,36]. These proven effects of compounds that are present in the 25% EtOH extract may have additional benefits in the treatment of wounds where inflammation, oxidation, modulation of signal transmission, and other mechanisms are important parts of a healthy healing process. Moreover, the stimulating effect of *Reynoutria* extract on fibroblasts, in addition to wounds caused by various injuries, surgical injuries, mouth ulcers and others, can also be used in periodontal diseases, where the proper division and activity of fibroblasts plays a key role [37,38]. The search for new non-toxic preparations facilitating oral wound healing seems justified, taking into account the results concerning the use of the most popular antiseptic—chlorhexidine. The commercial dental rinse (containing chlorhexidine digluconate 0.2%) used in this study was cytotoxic to HGFs even at the lowest concentration (1.56%). It did not stimulate division and migration of fibroblasts or collagen production. Other in vitro studies confirm the toxic effect of chlorhexidine on cells, including fibroblast, osteoblastic or endothelial cells, at concentrations far below those used in clinical practice [39,40]. Scientists are asking for more cautious use of chlorhexidine as a wound healing aid, especially as the evidence for the effectiveness of chlorhexidine in the treatment of certain oral ailments is rather unclear [14]. In addition to using new natural compounds to heal oral wounds in place of chlorhexidine, another way may also be the use of low doses of chlorhexidine together with natural substances [15]. Such combinations should aim to minimize side effects and maximize benefits.

## 4. Conclusions

The 25% EtOH, 40% EtOH, and 60% acetone extracts from rhizomes of *R. japonica* stimulated gingival fibroblasts to proliferate and migrate, as well as resulting in increased synthesis of collagen III. These results provide support for the traditional use of *R. japonica* rhizome as a gingival wound healing treatment. However, extracts differed in the strength of their activity. The 25% EtOH extract strongly stimulated fibroblasts proliferation and migration, which may be related to its highest content of resveratrol and an appropriate composition of procyanidins in it.

## Figures and Tables

**Table 1:** Wound (gap) closure percentage. Data obtained in ImageJ.

	Incubation Time (h)		
	24	48	72
untreated cells	3%	30%	50%
25% EtOH	20%	60%	85%
40% EtOH	7%	60%	80%
60% acetone	7%	50%	50%
commercial rinse	0%	15%	15%

**Table 2:** Evaluation of the intensity of immunocytochemical reaction in human fibroblasts using antibodies against collagen type III after 24 h exposure to different extracts of *Reynoutria japonica* (at 50 µg/mL) and commercial dental rinse (1.56% solution). Note that 25% EtOH, 40% EtOH, and 60% acetone means 25% ethanol extract, 40% ethanol extract, or 60% acetone extract from *Reynoutria japonica* rhizomes, respectively. Positive control: betulinic acid (2 µM).

Sample	The Intensity of Staining	Stained Cells (%)
untreated cells	−	96 ± 2
positive control	++	95 ± 1.5 *
25% EtOH extract	+/++	97 ± 3 *
40% EtOH extract	++/+++	98 ± 2 *
60% acetone extract	++	95 ± 1 *
commercial rinse	−	98 ± 1.5 *

* *p* < 0.05 with reference to control cells (Student’s *t*-test). The intensity of immunohistochemical staining was evaluated as (−) negative, (+) weak, (++) moderate, or (+++) strong.

**Table 3:** Retention times, UV λ max, MS data, and ion formula suggestion of the constituents present in the extracts of *R. japonica* rhizomes.

Nr.	Compound	Rt. (min)	UV max (nm)	*m*/*z* (M − H)^−^	Error (ppm)	Ion Formula	MS^2^ Main-Ion (Relative Intensity %)	MS^2^ Fragments (Relative Intensity %)	References
1	Unknown carbohydrate, e.g., sucrose	1.0	ND	341.1093	−1.0	C_12_H_21_O_11_	113.0235(100)	119(77),101(66), 173(25), 179(23)	HMDB0000258
2	Unknown carbohydrate	1.05	ND	719.2032	1.2	C_30_H_39_O_20_	377.0864(100)	379(27), 341(13), 179(10)	−
3	Unknown	1.1	ND	439.0815	1.9	C_26_H_15_O_7_	96.9622(100)	241(6)	−
4	Organic acid, e.g., malic acid	1.15	ND	133.0145	−2.2	C_4_H_5_O_5_	115.0031(100)		HMDB0000156
5	Organic acid, e.g., citric acid	1.3	ND	191.0200	−1.4	C_6_H_7_O_7_	111.0085(100)		HMDB0000094
6	Procyanidin dimer, Type B	1.6–1.8	225,280	577.1350	0.2	C_30_H_25_O_12_	289.0724(100)	407(60), 125(37), 425(16)	[23]
7	Procyanidin trimer, Type B	1.8–1.9	225,280	865.198	0.6	C_45_H_37_O_18_	575(100)	287(93), 577(80), 695(57)	[23]
8	Procyanidin dimer, Type B	1.9–2.0	225,280	577.1345	1.8	C_30_H_25_O_12_	289.0719(100)	407(69), 125(41), 425(22)	[23]
9	Procyanidin trimer, Type B	2.0–2.1	225,280	865.1988	−0.3	C_45_H_37_O_18_	577.1341(100)	287(92), 575(90), 865(75), 425(58), 289(45), 695(44), 713(42)	[23]
10	Catechin	2.1	225,280	289.0721	−1.1	C_15_H_13_O_6_	123.0451(100)	109(90), 125(73), 245(36)	[23]
11	Procyanidin dimer, Type B	2.2	225,280	577.1358	−1.1	C_30_H_25_O_12_	289.0723(100)	407(62), 125(33), 425(20)	[23]
12	Procyanidin dimer, Type B	2.4	225,280	577.1350	0.2	C_30_H_25_O_12_	289.0724(100)	407(60), 125(39), 425(15)	[23]
13	Epicatechin	2.8	225,280	289.0724	−0.8	C_15_H_13_O_6_	109.0293(100)	123(95), 221(81), 125(77)	[23]
14	Procyanidin trimer, Type B	3.3	225,280	865.2000	−1.7	C_45_H_37_O_18_	577.1340(100)	287(82), 575(75), 865(66), 425(54), 713(44), 413(42), 695(40)	[23]
15	Piceatannol glucoside	3.4	220,290,318	405.1197	−1.4	C_20_H_21_O_9_	243.0668(100)	244(14), 245(2), 201(1)	[16]
16	Procyanidin dimer monogallate	3.6	225,280	729.1448	1.8	C_37_H_29_O_16_	407.0787(100)	289(46), 577(29), 451(21)	[23]
17	Resveratrolside	3.8	220,304,315	389.1249	−1.8	C_20_H_21_O_8_	227.0716(100)	228(16), 225(9), 185(2)	[16]
18	Procyanidin dimer monogallate	4.1	225,280	729.1463	−0.2	C_37_H_29_O_16_	407.0776(100)	289(62), 577(45), 441(29), 451(27)	[23]
19	Procyanidin tetramer, Type B	4.6	225,280	1153.2605	1.3	C_60_H_49_O_24_			[23]
20	Unknown	5.5	220,275	269.0124	2.7	C_7_H_9_O_11_	189.0559 (100)		−
21	Piceid	6.0	220,304,315	389.1248	−1.6	C_20_H_21_O_8_	227.0715(100)	228(14), 185(2), 225(0.5)	[16]
22	Epicatechin-3-O-gallate	6.3	220,279	441.0829	−0.3	C_22_H_17_O_10_	169.0142(100)	289(55), 125(22), 245(13)	[16]
23	Procyanidin trimer monogallate	6.7–6.8	225,280	1017.2140	−4.4	C_52_H_41_O_22_	729.1431(100)	577(30), 865(29), 287(27), 441(19)	[23]
24	Procyanidin tetramer monogallate	7.3	225,280	652.1308 [M − 2H]^2−^, 1305.2683	−6.4	C_67_H_53_O_28_	125.0243(100)	169(96), 289(54), 407(26), 451(11), 577(7), 729(5)	[23]
25	Resveratrol hexoside	7.8	220,304,315	389.1242	−0.1	C_20_H_21_O_8_	227.0713(100)	228(15), 185(1), 143(0.5)	[16]
26	Resveratrol derivative	8.4	220,282,325	431.1349	−0.3	C_22_H_23_O_9_	227.0714(1000)	228(14), 185(1)	[16]
27	Resveratrol hexoside	8.7	220,304,315	389.1247	−1.3	C_20_H_21_O_8_	227.0716(100)	228(15), 185(2), 143(0.5)	[16]
28	Aloesone hexoside	9.0	220,280,420	393.1199	−2.0	C_19_H_21_O_9_	231.0664(100)	232(14),187(0.5)	HMDB35734
29	Emodin-glucoside	9.7	221,247,269,281,423	431.0989	−1.2	C_21_H_19_O_10_	431.0984(100)	269.0456(77)	[16]
30	Lapathoside D	10.0	220, 290, 315	633.1818	1.2	C_30_H_33_O_15_	145.0295(100)	487(43), 633(30)	[23]
31	Resveratrol	10.6	220,306,319	227.0716	−1.2	C_14_H_11_O_3_	143.0499(100)	185(82), 227(38)	[16]
32	Torachrysone- hexoside	12.3	226,266,325sh	407.1352	−1.1	C_20_H_23_O_9_	245.0823(100)	246(14), 230(11)	[16]
33	Emodin-glucoside	12.8	221,269,281, 423	431.0987	−0.9	C_21_H_19_O_10_	269.0459(100)	311(5)	[16]
34	Emodin-8-O-(6′-O-malonyl)-glucoside	14.2	220,281,423	517.0991	−0.7	C_24_H_21_O_13_	473.1087(100)	269(65), 311(4)	[16]
35	Unknown Torachrysone derivative	14.5	220,266,325sh	449.1457	−0.9	C_22_H_25_O_10_	245.0818(100)	246(14)	−
36	Torachrysone	14.8	220,312	245.0824	−1.8	C_14_H_13_O_4_	230.0589(100)	215(52)	[16]
37	Physcionin/Rheochrysin	15	221,272,423	445.1136	0.9	C_22_H_21_O_10_	283.0610(100)	284(17), 307(5)	HMDB0040511/HMDB35931
38	Emodin bianthrone-hexose-(malonic acid)-hexose	15.5	220,280,325	919.2318	−1.7	C_45_H_43_O_21_	458.1215(100)	416(49), 671(18), 713(14), 875(13)	[23]
39	Hydropiperoside	15.7	222,298,313	779.2188	0.7	C_39_H_39_O_17_	779.2176(100)	145(80),633(61), 453(6), 615(5)	[16]
40	Emodin bianthrone-hexose-(malonic acid)-hexose	16.1	220,280,325	919.2300	0.2	C_45_H_43_O_21_	458.1207(100)	416(50), 502(28), 713(18), 671(18), 875(16)	[23]
41	Derivative of Emodin bianthrone-hexose-malonic acid	16.4	220,280,325	1005.2321	−1.5	C_48_H_45_O_24_	458.1222(100)	502(27), 713(21), 917(11), 961(5)	[23]
42	Unknown physcion derivative	17	220, 275, 423	1063.2355	0.6	C_50_H_47_O_26_	283.0607(100)	325(13), 487(7)	−
43	Emodin bianthrone-hexose-(malonic acid)-hexose	17.1	220,280,325	919.2309	−0.6	C_45_H_43_O_21_	458.1214(100)	416(43), 502(25), 713(15), 671(15), 875(11)	[23]
44	Derivative of Emodin bianthrone-hexose-malonic acid	17.7	220,285,325	1019.2444	1.8	C_49_H_47_O_24_	458.1211(100)	502(23), 931(9), 460(4), 416(2)	[23]
45	Derivative of Emodin bianthrone-hexose-malonic acid	17.8	220,280,325	1005.2305	0.1	C_48_H_45_O_24_	458.1212(100)	502(27), 713(21), 917(9), 961(2)	[23]
46	Phenylpropanoid-derived disaccharide esters	18	220, 298,315	1151.3406	−0.4	C_59_H_59_O_24_	1151.3358(100)	955(20), 145(5), 1133(2), 1103(2), 1009(2), 809(2)	[23]
47	Vanicoside B isomer	18.8	222,298,315	955.2668	−0.2	C_49_H_47_O_20_	955.2670(100)	145(22), 809(19), 957(16), 453(2)	[23]
48	Vanicoside B	19.3	222,298,315	955.2662	0.4	C_49_H_47_O_20_	955.2665(100)	957(17), 809(15), 145(14), 453(1)	[16]
49	Questin	20.2	222,286,313, 430	283.0611	0.4	C_16_H_11_O_5_	240.0425(100)	268(3)	[16]
50	Vanicoside A	21.1	222,298,315	997.2757	1.5	C_51_H_49_O_21_	997.2776(100)	145(8), 851(5), 821(1)	[16]
51	Emodin bianthrone-hexose-malonic acid	21.9	220,280,325	757.1752	2.9	C_39_H_33_O_16_	458.1220(100)	502(8), 713(5), 254(4), 416(2)	[23]
52	Emodin bianthrone-hexose-malonic acid	22.6	220,280,325	757.1743	4.1	C_39_H_33_O_16_	458.1214(100)	502(10), 254(4), 713(4), 416(2)	[23]
53	Emodin	25.4	221,248,267, 288,430	269.0455	0.3	C_15_H_9_O_5_	269.0455(100)	225(29), 241(11), 197(2), 181(1)	[16]
54	Physcion *	29.5	222,266,288, 430	285.0757 [M − H]^+^	−1.9	C_16_H_13_O_5_ [M − H]^+^	−		[16]

HMDB ID: The Human Metabolome Database, Physcion*—observed only in the positive mode.

**Table 4:** Linearity, LOD, LOQ data for used standards.

Analyte	λ _det_ (nm)	Calibration Equation	*n*	r	Linear Range (μg/mL)	LOD (μg/mL)	LOQ (μg/mL)
Piceid	298	*y* = 32.83*x* − 7.9417	6	0.9999	11.25−360	5.29	16.02
Resveratrol	298	*y* = 60.021*x* − 0.4491	6	0.9998	1.25−40	1.04	3.16
Vanicoside B	298	*y* = 26.719*x* − 3.8342	6	0.9999	4.375−140	1.41	4.28
Vanicoside A	298	*y* = 34.161*x* − 2.9891	6	0.9995	0.9375−30	0.23	0.71
Emodin	298	*y* = 20.389*x* − 2.444	6	0.9999	4.375−140	1.04	3.15
Physcion	298	*y* = 7.1533*x* − 1.8287	6	0.9999	3.75−120	1.02	3.08

**Table 5:** Content of analyzed compounds in studied extracts (mg/g of raw extract dry mass).

Analyte	*R. japonica* 60% Acetone		*R. japonica* 40% EtOH		*R. japonica* 25% EtOH	
	(mg/g)	%CV	(mg/g)	%CV	(mg/g)	%CV
Piceid	47.88	0.65	39.12	0.57	26.11	0.18
Resveratrol	1.47	0.71	3.05	0.69	3.95	0.26
Vanicoside B	5.67	0.36	1.77	0.63	0.30 *	0.42
Vanicoside A	0.53	0.25	0.21	0.70	0.05 *	2.21
Emodin	9.63	0.34	3.68	0.35	1.47	0.34
Physcion	7.79	0.78	0.36 *	0.46	0.31 *	0.41

* level below LOQ but above LOD.

## Data Availability

The data presented in this study are available on request from the corresponding author.
